# A Dual-Dynamic Crosslinked Polysaccharide-Based Hydrogel Loaded with Exosomes for Promoting Diabetic Wound Healing

**DOI:** 10.3390/ma19020445

**Published:** 2026-01-22

**Authors:** Ding Lin, Zhenhao Li, Jianying Hao, Xiaobo Xu, Xiuqiang Li, Yuan Feng, Xiaochen Lu, Fanglian Yao, Hong Zhang, Junjie Li

**Affiliations:** 1 School of Chemical Engineering and Technology, Tianjin University, Tianjin 300350, China; abc120010_@tju.edu.cn (D.L.);; 2 School of Science, Tianjin University, Tianjin 300350, China; 3 State Key Laboratory of Synthetic Biology, Tianjin University, Tianjin 300350, China; 4 Frontiers Science Center for Synthetic Biology (Ministry of Education), Tianjin University, Tianjin 300350, China

**Keywords:** hydrogel, diabetic wound, reactive oxygen species (ROS), exosomes

## Abstract

Diabetic wounds are often accompanied by severe inflammation, which is unfavorable for vascular growth and wound repair. Therefore, promoting the healing of diabetic wounds is of great significance. In this study, carboxymethyl chitosan (CMCS) was grafted with 4-formylphenylboronic acid (FPBA) and then crosslinked with oxidized sodium alginate (OAlg) to form a dual-dynamic covalent hydrogel (CPOA) based on borate ester bond and Schiff base bonds. Mesenchymal stem cells’ exosomes (Exos) were incorporated into the CPOA to construct CPOA@Exos for diabetic wound healing. Owing to the dual-dynamic covalent crosslinking network, the CPOA hydrogel showed good injectability and self-healing ability. In addition, the hydrogel displayed reactive oxygen species (ROS) responsive properties, enabling both scavenging of multiple free radicals and on-demand release of Exos in the ROS-rich wound microenvironment. A diabetic wound model was established on C57 mice, and treatment with CPOA@Exos demonstrated that it could promote the polarization of macrophages toward the M2 phenotype, enhance cellular proliferation in the wounded area, and thereby accelerate the healing of diabetic wounds. In conclusion, this study provides a new hydrogel wound dressing that can inhibit inflammation for the management of diabetic wounds.

## 1. Introduction

Diabetes mellitus has affected over 400 million individuals worldwide [1], and the global prevalence of diabetes among adults is projected to reach 7.7% by 2030 [2]. Diabetic wounds, a common complication of diabetes, affect approximately 25% of diabetic patients [3], posing a significant public health threat to human health.

Normal wound healing involves four sequential phases: hemostasis, inflammation, proliferation, and remodeling [4]. Diabetic wounds, however, present pathological hallmarks (hyperglycemia, chronic inflammation and excessive reactive oxygen species [ROS] [5,6]), which trap the wounds in a prolonged inflammatory state, impeding healing progression. Excessive ROS induces damage to cellular components, proteins, and DNA, exacerbating tissue injury [7], while sustained inflammation suppresses extracellular matrix formation, delaying wound closure [8]. Notably, local immune dysfunction is the core driver of excessive inflammation [9,10], with macrophages serving as key regulators [11]. In normal healing, macrophages polarize to the pro-inflammatory M1 phenotype (early phase [12]) and the pro-healing M2 phenotype (late phase [13]). However, in diabetic wounds, the persistence of M1 macrophages impedes M2 activation, resulting in chronic inflammation [14]. Thus, modulating the M1/M2 phenotypic polarization of macrophages and scavenging excessive ROS are critical therapeutic targets for diabetic wound treatment [15].

Current clinical management of diabetic wounds primarily relies on conventional dressings such as gauze. While these provide a basic physical barrier, their repeated removal may cause adhesive trauma to the wound [16]. Hydrogel dressings, by contrast, are promising alternatives, as they possess physiological water content [17], good biocompatibility, and the ability to maintain a moist wound microenvironment—all of which favor healing [18]. To adapt to the irregular topography of diabetic wounds, hydrogels must exhibit robust adhesive properties [19]. The Schiff base reaction (between aldehyde and amino groups) is a widely used hydrogel crosslinking strategy [20], endowing hydrogels with self-healing capabilities [21] and strong tissue adhesion—mediated by the Schiff reaction between aldehyde groups and tissue-derived amino groups [22,23]. However, diabetic wounds are often accompanied by severe oxidative stress [24], and the hydrogels, despite maintaining a moist environment, fail to effectively promote wound repair. To address this limitation, integrating stimulus-responsive moieties into hydrogel networks has emerged as a promising approach, enabling the hydrogels to respond to disease-specific microenvironments, achieve controlled release of loaded drugs, and optimize therapeutic efficacy [25]. Specifically, the incorporation of dynamic borate ester bonds into the gel network confers ROS-responsive properties, as these bonds can dissociate upon exposure to ROS [26], and even scavenge a portion of ROS, thereby reducing oxidative stress at the wound site [27]. Combining Schiff base (adhesion/self-healing) and borate ester (ROS responsiveness) bonds fabricates multifunctional hydrogels ideal for diabetic wounds [28,29].

Drug loading in hydrogels is an effective strategy to promote diabetic wound repair [30]. Exosomes from mesenchymal stem cells (Exos) are nanosized vesicles (30–150 nm in diameter) secreted by cells, containing various proteins, lipids, and nucleic acids, which play a crucial role in intercellular communication [31]. Numerous studies have demonstrated the Exos enriched with bioactive growth factors can induce M2 macrophage polarization through multiple pathways, such as the integrin β3/SOCS3/STAT3 pathway [32] and S1P/SK1/S1PR1 signaling pathway [33], thereby regulating tissue inflammation and immune responses. However, direct application of Exos to wounds suffers from rapid clearance, limiting their therapeutic efficacy [34]. In contrast, loading Exos into hydrogels can enhance their stability and enable sustained release, thereby improving their therapeutic effects in diabetic wound treatment [35,36].

**Scheme 1:**
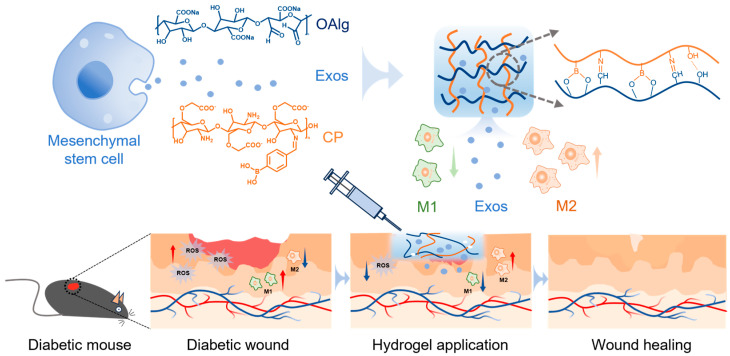
Schematic of the dual-dynamic crosslinked hydrogel structure and its repair effect on diabetic wounds (OAlg: oxidized sodium alginate, Exos: exosomes, CP: carboxymethyl chitosan-grafted phenylboronic acid, M1: M1 macrophages, M2: M2 macrophages).

Dual-dynamic crosslinking systems based on Schiff base and borate ester chemistry have been reported in the literature to enhance the therapeutic efficacy of diabetic wounds [5]. In contrast, this study is distinguished by the innovative design of a modified polysaccharide-based dual-crosslinked network hydrogel (CPOA) composed of carboxymethyl chitosan-grafted phenylboronic acid (CP) and oxidized sodium alginate (OAlg) based on Schiff base and borate ester bonds and its synergistic combination with Exos (CPOA@Exos) to accelerate wound healing (Scheme 1). Specifically, 4-formylphenylboronic acid (FPBA) was grafted onto carboxymethyl chitosan (CMCS) to obtain a series of FPBA-grafted CMCS derivatives. The CP was then mixed with OAlg to form the CPOA hydrogel. In a weakly alkaline microenvironment, the boronic acid groups of CP reacted with cis-diol units on OAlg to form borate ester bonds. Simultaneously, the amino groups in CP formed Schiff base bonds with the aldehyde groups in OAlg, collectively resulting in a dual dynamic covalent crosslinked hydrogel network. This modified polysaccharide matrix provides excellent biocompatibility, making it an ideal carrier for Exos loading. Moreover, the borate ester bonds impart ROS-responsive properties to the hydrogel, allowing on-demand release mechanism that enables the Exos to fully exert their anti-inflammatory and wound-repair-promoting effects. Beyond this, this system integrates the intrinsic antioxidant capacity of the hydrogel with the biological functions of Exos, resulting in a synergistic therapeutic effect that effectively promotes diabetic wound repair. Additionally, this study evaluated the mechanical properties, self-healing behavior, and adhesion performance of the CPOA hydrogels, and the *in vivo* repair efficacy was assessed using a diabetic wound model in C57 mice.

## 2. Materials and Methods

### 2.1. Materials

Sodium alginate (Alg), hydrogen peroxide (H_2_O_2_) and 1,1-diphenyl-2-trinitrophenylhydrazine (DPPH) were purchased from Aladdin Co., Ltd. (Shanghai, China). Carboxymethyl chitosan (CMCS) was supplied by Yuanye Bio-Technology Co., Ltd. (Shanghai, China). 4-formylphenylboronic acid (FPBA), Sodium hydroxide (NaOH) and sodium periodate (NaIO_4_) were sourced from Meryer Chemical Technology Co., Ltd. (Shanghai, China). Hydrochloric acid (HCl) was obtained from Fengchuan Chemical Reagent Co., Ltd. (Tianjin, China). Ethylene glycol was purchased from Heowns Biochem Technologies LLC (Tianjin, China). Exosomes (Exos) were acquired from Mingyang New Materials Co., Ltd. (Tianjin, China). The ABTS assay kit was provided by Beijing Bio-Ocean Technology Co., Ltd. (Beijing, China). RPMI-1640 basal medium and the CCK-8 cell proliferation assay kit were purchased from Beijing Solarbio Technology Co., Ltd. (Beijing, China). All chemicals were used as received without further purification unless otherwise stated.

### 2.2. Preparation of Oxidized Sodium Alginate

First, 5 g of Alg was dissolved in 25 mL of anhydrous ethanol, while 1 g of NaIO_4_ was dissolved in 25 mL of water. The two solutions were then mixed under stirring for 4 h. The reaction was then terminated by adding 2 mL of ethylene glycol. The resulting mixture was dialyzed against water for 3 days and subsequently freeze-dried to yield OAlg. The oxidation degree (*OD*) of the resulting OAlg was quantified by hydroxylamine hydrochloride titration. Specifically, 0.1 g of OAlg was dissolved in 25 mL of hydroxylamine hydrochloride-methyl orange solution. After stirring for 2 h, the mixture solution was titrated with a 10 mmol/L NaOH solution, and the released HCl was measured using potentiometric titration. The *OD* of the OAlg was calculated according to Equation (1):

(1) $$OD(\%)=\frac{0.001\times c(NaOH)\times ∆V/2}{w(OAlg)/198.11}\times 100$$

Whereas, *c*(*NaOH*) is the concentration of NaOH solution, ∆*V* represents the volume of NaOH solution consumed, and *w*(*OAlg*) is the mass of the OAlg.

### 2.3. Preparation of 4-Formylphenylboronic Acid Grafted Carboxymethyl Chitosan

CMCS was dissolved in 80 mL of water. Varying amounts of FPBA (0.08, 0.16, 0.24 and 0.32 g) were then added into the CMCS solution, respectively. The mixtures were stirred for 6 h, and the resulting solutions were purified by precipitation with anhydrous ethanol. The collected solids were dried to obtain a series of FPBA-grafted CMCS derivatives, which were designated as CP1, CP2, CP3 and CP4, respectively, based on the substitution degree of FPBA on the CMCS chain. The chemical structure of the prepared CP was confirmed by nuclear magnetic resonance (^1^H NMR) spectroscopy (Bruker, Billerica, MA, USA, Avance NEO, 800 MHz) and Fourier transform infrared (FTIR) spectroscopy (FEI, Hillsboro, OR, USA, iS50).

### 2.4. Preparation of Dual-Dynamic Crosslinked Hydrogel Loaded with Exosomes

First, 6% (*w*/*v*) OAlg, CMCS, CP1, CP2, CP3 and CP4 solutions in PBS were prepared separately. To investigate how the CP2-to-OAlg volume ratio affects hydrogel properties and to identify the optimal formulation, OAlg and CP2 solutions were mixed in a centrifuge tube at a series of volume ratios (3:7, 4:6, 5:5, 6:4 and 7:3) using vortex oscillation. This preparation method yielded hydrogels with varying compositions. Adjusting this ratio allows direct modulation of the crosslinking degree of both Schiff base and borate ester bonds, which in turn regulates the hydrogel’s structure and overall properties (particularly its mechanical properties), a critical factor for subsequent applications. Then OAlg solution was mixed with CMCS, CP1 and CP2 separately at a 1:1 volume ratio to fabricate a dually dynamically cross-linked injectable hydrogel, designated COA, CP1OA and CP2OA. The specific formulations are listed in Table S1. The network of the CPOA hydrogel was investigated via Scanning Electron Microscope (SEM) (FEI, Hillsboro, OR, USA, Apreo S LoVac). The effect of the FPBA substitution degree on gelation formation was thoroughly investigated. Based on these results, exosomes derived from mesenchymal stem cells were incorporated into the CPOA hydrogel, yielding an exosome-loaded dually dynamically cross-linked hydrogel (CPOA@Exos). The morphology of exosomes was observed using transmission electron microscopy (TEM) (JEOL, Tokyo, Japan, JEM-2100F).

### 2.5. Rheological Properties Test

The rheological properties of the CPOA hydrogels were evaluated using a rotational rheometer (Anton Paar, Graz, Austria, MCR 302e). Approximately 400 μL of the hydrogel sample was placed on the instrument plate, and the measuring gap was set to 1.0 mm at 25 °C. The test sequence was as follows: first, a time sweep was performed at a fixed frequency of 1.0 Hz and a strain of 1% to characterize the storage modulus (*G*′) and loss modulus (*G*″). Subsequently, a strain amplitude sweep from 1% to 1000% was conducted at a constant frequency of 1.0 Hz to determine the critical strain at which the hydrogel failed. Finally, the hydrogel’s viscosity was examined under shear rates ranging from 0.001 s^–1^ to 10 s^–1^.

### 2.6. Injectability and Self-Healing Property Evaluation

The injection force of the hydrogels was evaluated using a 1 mL dual-barrel syringe with a 21-gauge needle mounted on a universal testing machine (SPAI, Jinan, China, WDW-T05). The syringe was compressed at a rate of 10 mm/min, and the changes in force-displacement were recorded.

The self-healing capability of the CPOA hydrogels was evaluated through macroscopic and rheological assessments. For the macroscopic test, two CPOA hydrogels with different colors (pink and colorless) were prepared, each cut into two halves. The segments from different hydrogels were then tightly merged and incubated at room temperature for 1 h to assess the interfacial integration. Subsequently, a rotational rheometer (MCR 302e) was employed to conduct a cyclic strain test, alternating between low strain (1%, 50 s) and high strain (400%, 50 s) at a fixed frequency of 1.0 Hz. The *G*’ and *G*″ modulus were recorded to quantify the healing behavior.

### 2.7. Adhesion Performance Test

The adhesion properties of the prepared CPOA hydrogels were evaluated via lap shear tests. A total of 200 μL of hydrogel was applied onto a rectangular pig skin with a width of 20 mm, resulting in an overlap area of 2 cm^2^. Another piece of pig skin was then placed over it to ensure complete contact. The skin-hydrogel samples were clamped to the jaws of a universal tensile testing machine and subjected to tensile separation at a crosshead speed of 20 mm/min to measure the adhesive strength.

### 2.8. Degradation In Vitro Test

To investigate the degradation behavior, the hydrogels were immersed in PBS and H_2_O_2_ (500 μmol/L) solutions at 37 °C. The immersion solutions were refreshed, and the hydrogels were taken out to record their morphological changes every 24 h. After 7 days, all the liquids were removed, and the remaining hydrogels were freeze-dried and weighed. The mass retention rate at different time points was calculated using Equation (2),

(2) $$Retention\;rate\;\left(\%\right)=\frac{w_{t}}{w_{0}}\times 100$$

Whereas, *w*_0_ and *w*_t_ refer to the initial mass and the freeze-dried mass at time *t*, respectively.

### 2.9. Measurement of Exosomes Release Profile

To minimize interference from hydrogel degradation products during exosomes release measurement, 100 μL of CPOA@Exos was loaded into a dialysis bag (MWCO: 8000–14,000). The bag was then immersed in 500 μL PBS or H_2_O_2_ (500 μmol/L) solutions and incubated at 37 °C. At predetermined time intervals, 20 μL of the supernatant was collected and replaced with an equal volume of the corresponding fresh solution. The released Exos were quantified using a BCA protein assay kit (Beyotime Biotechnology, Shanghai, China). Briefly, reagents A and B from the BCA kit were mixed at a ratio of 50:1. Subsequently, 200 μL of the prepared BCA working solution was added to each well, and the plate was incubated at 37 °C for 1 h. The absorbance at 562 nm was measured using a microplate reader (Tecan, Männedorf, Switzerland). The standard curve was generated using the Exos standard data, and the Exos release curve was derived from this. The release rate of Exos from the CPOA hydrogel was calculated according to Equation (3)

(3) $$Exos\;release\;rate\left(\%\right)=\frac{A_{t}-b}{A_{1}-b}\times 100$$

Whereas *b* represents the intercept of the standard curve, *A_t_* represents the absorbance of 20 μL of supernatant mixed with 200 μL of BCA solution at time *t*, and *A*_1_ represents the absorbance of Exos (1/6 × 10^9^ particles/mL) mixed with 200 μL of BCA solution.

### 2.10. Measurement of Antioxidant Properties

The antioxidant properties of hydrogels were evaluated by measuring their free radical scavenging activities against ABTS·^+^, DPPH· and hydroxyl radicals. For the ABTS·^+^ scavenging assay, a stock solution was prepared by mixing 7 mmol/L ABTS·^+^ with an equal volume of 2.45 mmol/L potassium persulfate solution. The mixture was allowed to react in the dark at 4 °C for 12 h before use. The stock solution was then diluted with water to achieve an absorbance of 0.7 ± 0.02 at 734 nm, thus obtaining the ABTS·^+^ working solution. Subsequently, 200 μL of CPOA hydrogel was added to 1 mL of ABTS·^+^ working solution. The mixture was incubated in the dark for 10 min, and the absorbance (*A*_1_) was measured at 734 nm. For the control group, an equal volume of water was used instead of the hydrogel, and its absorbance was recorded as *A*_0_. The ABTS·^+^ scavenging effect was calculated using Equation (4)

(4) $${ABTS\cdot }^{+}scavenging\;effect\;(\%)=\frac{A_{0}-A_{1}}{A_{0}}\times 100$$

For the DPPH· scavenging assay, 200 μL of CPOA hydrogel was added into 1 mL of 0.1 mmol/L DPPH ethanol solution. The mixture was incubated in the dark for 30 min, and the absorbance was measured at 517 nm (*A*_1_). Two controls were established: in the blank group, the DPPH solution was replaced with ethanol, while in the negative control, the hydrogel was replaced with water. Their absorbances were designated as *A*_2_ and *A*_0_, respectively. The DPPH· scavenging effect was determined according to Equation (5).

(5) $$DPPH\cdot scavenging\;effect\;(\%)=(1-\frac{A_{1}-A_{2}}{A_{0}})\times 100$$

For the ·OH scavenging assay, a reaction mixture was prepared in a test tube by adding 0.3 mL of 10 mmol/L salicylic acid solution, 0.3 mL of 10 mmol/L ferrous sulfate solution and 0.3 mL of 8.8 mmol/L hydrogen peroxide solution. Then, 200 μL of CPOA hydrogel was soaked in the mixture solution and incubated in the dark at 37 °C for 30 min. After incubation, the absorbance of the solution was measured at 510 nm (*A*_1_). In the blank control, salicylic acid solution was replaced with an equal volume of water, and in the negative control, the hydrogel was replaced with an equal volume of water. The corresponding absorbance values were recorded as *A*_2_ and *A*_0_, respectively. The ·OH scavenging effect was determined according to Equation (6).

(6) $$\cdot OH\;scavenging\;effect\;(\%)=(1-\frac{A_{1}-A_{2}}{A_{0}})\times 100$$

### 2.11. Hemocompatibility Test

Red blood cells (RBCs) were isolated from anticoagulated rabbit blood (Solarbio, Beijing, China) by centrifugation at 1500 rpm for 10 min and subsequently diluted to 10% (*v*/*v*) suspension. Meanwhile, 400 μL of CPOA hydrogel was immersed in 4 mL of PBS at 37 °C for 24 h to prepare the hydrogel extract. Then, 0.9 mL of the extract was mixed with 0.1 mL of RBC suspension and incubated at 37 °C for 4 h. After centrifugation at 1500 rpm for 5 min, the absorbance of the supernatant was measured at 541 nm. In the control, PBS and water were used to replace the extract as the negative and positive controls, respectively. The hemolysis rate was calculated using Equation (7), where *A_positive_*, *A_negative_* and *A_sample_* represent the absorbance of the positive control, negative control, and hydrogel group, respectively.

(7) $$Hemolysis\;rate\;\left(\%\right)=\frac{A_{sample}-A_{negative}}{A_{positive}-A_{negative}}\times 100$$

### 2.12. Cytotoxicity Assay

The compatibility of the hydrogel was evaluated using L929 cells via the CCK-8 assay and acridine orange (AO) (Baiao Leibo, Beijing, China) fluorescence staining. The CPOA hydrogel extract was obtained by immersing 1 mL of hydrogel in 10 mL of RPMI 1640 medium for 24 h. Meanwhile, L929 cells were seeded in 96-well plates at a density of 5000 cells/well. After being cultured for 12 h, the culture medium was replaced with hydrogel extract, and the cells were incubated for another 24 h or 48 h. Cells incubated with RPMI 1640 medium instead of the extract served as the negative control. Cell viability was determined using a CCK-8 kit according to the manufacturer’s protocol and was calculated using Equation (8), where *A_control_*, *A_blank_*, and *A_sample_* represent the absorbance of the control group, the blank group (medium without cells) and the hydrogel group, respectively.

(8) $$Cell\;viability\;\left(\%\right)=\frac{A_{sample}-A_{blank}}{A_{control}-A_{blank}}\times 100$$

In addition, AO staining was employed to assess cell viability and cell morphology. After washing with PBS, the cells were incubated with 100 μL of AO solution at 37 °C for 20 min. Following incubation, the cells were washed three times with PBS to remove excess dye. Cell morphology was observed under a fluorescence microscope (ECLIPSE Ts2, Nikon, Tokyo, Japan).

### 2.13. Scratch Wound Healing Assays

L929 cells were seeded in a 24-well plate and incubated for 24 h. A straight scratch wound was then created on the monolayer using a pipette tip. After washing with PBS, 500 μL of culture medium was added to each well, followed by the addition of 100 μL of CPOA or CPOA@Exos hydrogel. The cells were further incubated for 24 h, after which phase-contrast images were captured. The wound area was measured using ImageJ (version 1.54r) software, and the cell migration rate was calculated according to Equation (9), where *S*_0_ represents the initial scratch area and *S*_a_ denotes the scratch area after 24 h.

(9) $$Migration\;rate\;\left(\%\right)=\frac{S_{0}-S_{a}}{S_{0}}\times 100$$

### 2.14. Cell Proliferative Behaviors

To evaluate the cell proliferation-promoting effect of the CPOA@Exos hydrogel, a cell proliferation assay was performed using L929 cells. Specifically, the cells were seeded in 48-well plates at a density of 3000 cells/well. After culturing for 12 h, 50 μL of CPOA, 50 μL of CPOA@Exos, or 5 μL of Exos with 45 μL PBS were added to the corresponding wells, respectively. The cells were then co-cultured with these materials for 48 h and 120 h. At each time point, the morphology of cells was observed under a microscope first; subsequently, the cell viability was detected using a CCK-8 cell proliferation kit, and was calculated using Equation (8), where *A_control_*, *A_blank_*, and *A_sample_* represent the absorbance of the control group at 48 h, the blank group (medium without cells) and the hydrogel group, respectively.

### 2.15. Establishment of a Diabetic Skin Wound Model

All animal experiments were approved by Animal Ethical and Welfare of Tianjin University (Approval No.: TJUE-2024-138). Type 1 diabetes was induced in C57BL/6 mice (male, weighing 20–25 g) through selective destruction of pancreatic β-cells using streptozotocin (STZ). Briefly, after a 16 h fast, the mice received a single intraperitoneal injection of STZ. Following the injections, the drinking water was replaced with a 7% glucose solution for 2 h. Three days post-injection of STZ, blood glucose levels were monitored daily for consecutive days. Mice with blood glucose levels exceeding 16.8 mmol/L were considered diabetic. Subsequently, an 8 mm full-thickness skin defect model was created on the back of the prepared diabetic mice. The wounds were treated with 200 μL of PBS, CPOA, Exos (10^9^ particles/mL) and CPOA@Exos, respectively. Wound healing progression was documented using digital photography on days 0, 3, 7 and 14. The wound areas were recorded and quantified using ImageJ software, and the percentage of wound closure was calculated according to Equation (10), where *S*_0_ represents the initial wound area and *S_t_* denotes the wound area at day 3, 7 and 14.

(10) $$Healing\;rate\;\left(\%\right)=(1-\frac{S_{t}}{S_{0}})\times 100$$

### 2.16. Histochemical and Immunofluorescence Staining

At designated time points post-treatment, the mice were euthanized. Full-thickness skin tissues were harvested and fixed in 4% paraformaldehyde. For hematoxylin and eosin (H&E) staining (Solarbio, Beijing, China), deparaffinized sections were treated sequentially with hematoxylin (2 min), differentiation (10 s), water rinse, eosin (15 s), dehydration in ethanol, and mounting. For Masson’s trichrome staining (Solarbio, Beijing, China), sections were stained with Weigert’s hematoxylin (5 min), differentiated, blued (3 min), ponceau acid fuchsin (4 min), phosphomolybdic acid (1 min), aniline blue (10 s), rinsed between steps, dehydrated, and mounted. Images were acquired using a microscope (ECLIPSE Ts2, Nikon, Japan).

Additionally, immunofluorescence staining was performed to assess inflammation and angiogenesis through the healing process. Briefly, on days 3 and 7, the tissue sections were stained for CD86 and CD206 (1:400, Affinity Biosciences, Cincinnati, OH, USA) to observe macrophage polarization; On day 14, sections were stained for PCNA (1:400, Affinity Biosciences) to evaluate cell proliferation activity. Furthermore, immunohistochemical staining for IL-10 and TNF-α (1:400, Affinity Biosciences) was conducted on tissue sections on day 7 to assess the inflammatory cytokine expression levels during wound healing. Finally, the sections were examined under a fluorescence microscope (ECLIPSE Ts2, Nikon, Japan), and images were captured for further analysis. The ImageJ software was adopted for analyzing and calculating the cell fluorescence intensity.

### 2.17. Statistical Analysis

Each experiment was repeated at least three times (*n* ≥ 3). The experimental data are presented as mean ± standard deviation. The experimental data were statistically analyzed using Origin (version 2018) and Excel software. Statistical significance is indicated as follows: * *p* < 0.05, ** *p* < 0.01, *** *p* < 0.001, **** *p* < 0.0001.

## 3. Results and Discussion

### 3.1. Formation and Property of the Dual Dynamic Cross-Linked Hydrogel

**Figure 1:**
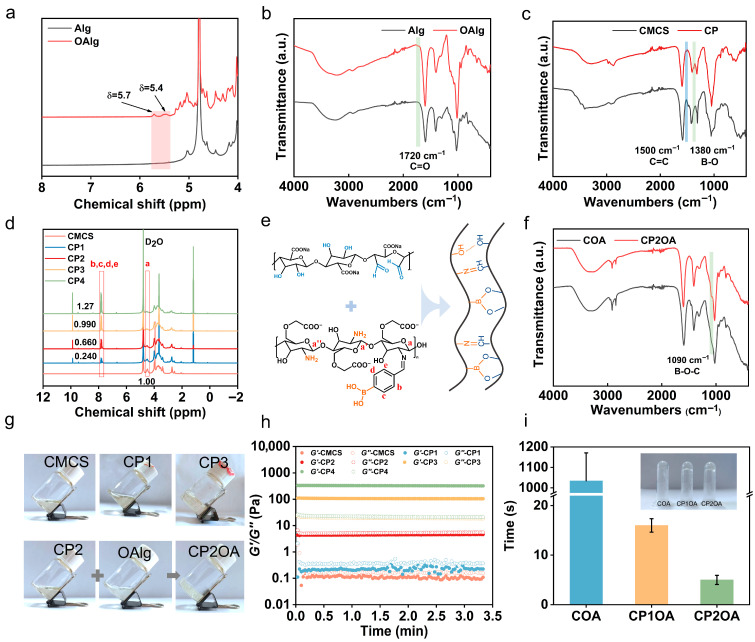
Formation and property of the dual dynamic cross-linked CPOA hydrogel. (**a**) ^1^H NMR spectra of Alg and OAlg; (**b**) FT-IR spectra of Alg and OAlg; (**c**) FT-IR spectra of CMCS and CP; (**d**) ^1^H NMR spectra of CMCS, CP1, CP2, CP3 and CP4; (**e**) The chemical structure and crosslinked structures of OAlg and CP; (**f**) FT-IR spectra of COA and CP2OA; (**g**) Spontaneous gelation of CP3 and hydrogel formation process of CP2OA; (**h**) Time sweep test (frequency = 1 Hz, strain = 1%) of CMCS, CP1, CP2, CP3 and CP4; (**i**) Gelation time of COA, CP1OA and CP2OA.

To prepare a dual-crosslinked network hydrogel with ROS-responsive properties, OAlg was synthesized by periodate oxidation of alginate using sodium periodate. As shown in Figure 1a, new chemical shifts at 5.4 and 5.6 ppm appeared in the ^1^H NMR spectra of OAlg compared with Alg, indicating the formation of a hemiacetal structure from the aldehyde group. In the FT-IR spectra, OAlg exhibited a new C=O stretching vibration at 1720 cm^–1^ (Figure 1b), which was absent in the spectrum of Alg. These results confirmed the successful synthesis of OAlg. The oxidation degree of OAlg was 14.74 ± 2.52%, measured by hydroxylamine hydrochloride titration. Meanwhile, FPBA was grafted onto CMCS chain via the amino groups of CMCS and the formyl groups of FPBA, yielding FPBA-grafted CMCS derivatives (CP). The substitution degree of FPBA could be modulated by varying the amount of FPBA. As shown in Figure 1c, compared to CMCS, the FT-IR spectra of CP exhibited new absorption bands at 1500 cm^−1^ and 1380 cm^−1^, which are assigned to the C=C stretching vibration of the benzene ring and the B-O stretching vibration from the boric acid moiety, respectively. Furthermore, by comparing the ^1^H NMR spectra of CMCS with CP1, new chemical shifts were observed in the range of 7.6–7.9 ppm (Figure 1d), which corresponded to the proton resonances of the benzene ring, indicating the successful synthesis of CP. The relative ratio of the integral area of the proton peaks (corresponding to carbons b, c, d and e) (Figure 1e) at 7.6–7.9 ppm to that of the proton peak associated with carbon a in CMCS at 4.31–4.61 ppm was used to calculate the substitution degree of FPBA, which were 6.00%, 16.50%, 24.75% and 31.75% for CP1, CP2, CP3 and CP4, respectively.

When CP and OAlg solutions were mixed, a dynamic cross-linked network was formed through the formation of Schiff base bonds between amino and aldehyde groups, as well as the creation of borate ester bonds between the adjacent hydroxyl groups on the G units of OAlg and the boronic acid structures in CP [37] (Figure 1e). The pH of the OAlg solution and CP2 solution was measured using a calibrated pH meter and determined to be 6.74 and 8.53, respectively. Upon mixing, the solution rapidly transitioned from a flowable liquid to a gel state. When the prepared hydrogel was immersed in a culture medium (pH 7.4), no detectable change in the medium pH was observed. The formation of dynamic covalent bonds was evidenced by the FT-IR spectra in Figure 1f, in which both COA and CP2OA hydrogels exhibited a decreased intensity in the C=O vibrational absorption compared with OAlg, suggesting the formation of Schiff base bonds. In addition, the spectrum of CP2OA revealed the asymmetric stretching vibration of B-O-C bond at 1090 cm^−1^, indicating the formation of borate ester bonds. Moreover, hydrogen bonding among the boric acid moieties of CP was also a key contributor to the hydrogel network formation. Especially, with the increase in the substitution degree of FPBA, both the *G*’ and *G*″ increased. When the substitution degrees of FPBA on CMCS exceeded 24.75%, the 6% CP3 and CP4 solutions spontaneously formed hydrogels (Figure 1g), with their *G*’ exceeding their *G*″ (Figure 1h). This gelation is likely attributable to the fact that once the substitution degrees of boronic acid groups from the grafted FPBA reached a critical level, they readily formed an extensive hydrogen-bond network with other functional groups such as amino and hydroxyl groups [38]. Based on these results, CP1OA and CP2OA were selected to fabricate injectable hydrogels with a dual dynamic crosslinked network. The substitution degree of FPBA was the main factor governing hydrogel formation. CP2OA exhibited the fastest gelation, requiring only 5.00 ± 0.71 s, compared to 1034.00 ± 137.64 s for COA (Figure 1i).

### 3.2. The Rheological and Adhesive Properties

**Figure 2:**
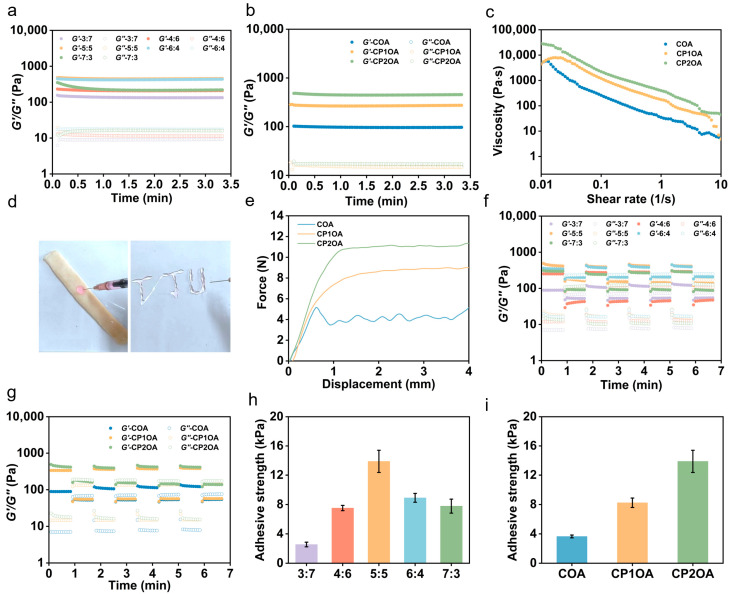
Rheological and adhesive properties of the hydrogels: (**a**) Time sweep test of hydrogels with different ratios; (**b**) Time sweep test of COA, CP1OA and CP2OA; (**c**) Shear-thinning behavior of COA, CP1OA and CP2OA; (**d**) Injectability demonstration; (**e**) Injection force of COA, CP1OA and CP2OA; (**f**) Cyclic strain test of hydrogels with different ratios (low strain = 1%, high strain = 400%); (**g**) Cyclic strain test of COA, CP1OA and CP2OA (low strain = 1%, high strain = 400%); (**h**) Lap shear test of hydrogels with different ratio; (**i**) Lap shear test of COA, CP1OA and CP2OA.

As the primary structural components, the dynamic Schiff base and borate ester bonds directly dictated the mechanical properties of the CPOA hydrogels. The CP/OAlg ratio and the FPBA substitution degree were thus identified as critical parameters for modulating the network structure and its mechanical properties. As shown in Figure 2a, the *G*’ of all the CPOA hydrogels exceeded their *G*″, confirming the formation of a stable gel network. The *G*’ value was only 136.38 ± 14.88 Pa at a CP2/OAlg ratio of 3:7, but increased with the OAlg content, reaching a maximum of 451.72 ± 28.82 Pa at a ratio of 5:5. Furthermore, variations in the substitution degree of FPBA also significantly influenced the structure and properties of CPOA hydrogels. SEM images showed that the hydrogel’s network structure became denser as the FPBA substitution degree in the CPs increased (Figure S1). Consistent with the trend, time sweep tests showed a corresponding increase in *G*’, with values of 97.07 ± 15.02, 269.54 ± 37.61 and 451.72 ± 28.82 Pa for COA, CP1OA and CP2OA, respectively (Figure 2b), further confirming the enhancement of mechanical properties with a higher FPBA substitution degree. In the strain amplitude sweeps, the *G’* of different hydrogels was greater than the *G*″ under low strain conditions. With the increase in strain, the curves of *G*’ and *G*″ intersected, indicating the destruction of the hydrogel network structure. Notably, all hydrogel groups could withstand a strain exceeding 100% (Figure S2a,b), which is conducive to their tight adhesion to the wound site.

Owing to the dual dynamic crosslinked network, the CPOA hydrogel showed good self-healing property and injectability. As shown in Figure 2c and Figure S2c, the CPOA hydrogels exhibited good shear thinning properties regardless of the composites, with viscosity decreasing as shear rate increased. Therefore, the hydrogel can be injected through a syringe fitted with a 21-gauge needle and subsequently shaped into a stable “TJU”-shaped gel (Figure 2d). The injection force was dependent on the FPBA substitution degree. The COA hydrogel exhibited an injection force of 4.22 ± 0.43 N, which increased with the formation of borate ester crosslinks. Notably, the injection force of CP2OA reached 10.98 ± 0.20 N (Figure 2e). Despite these values, they allowed for easier handling during manual operation and helped minimize the risk of surgical damage [39]. The dynamic crosslinking nature of borate esters and Schiff bases also endowed the CPOA hydrogel with good self-healing properties. As demonstrated by continuous alternating strain tests, under high strain (400%), the hydrogel structure underwent disrupted (*G*″ > *G*’), whereas returning to low strain (1%), the dynamic crosslinked network recovered (*G*’ > *G*″). Notably, after three consecutive cycles, *G*’ exhibited no significant change (Figure 2f,g). Moreover, after being cut and physically reconnected, the hydrogel rapidly restored its original structural integrity, owing to the reversible nature of the dynamic bonds (Figure S3a).

Upon injection into the wound surface, the aldehyde groups and phenylboronic acid groups present in CPOA hydrogels formed Schiff base bonds and hydrogen bonds with amino groups on the skin surface, resulting in strong adhesion to pig skin and various animal organs (Figure S3b). Bonding tests indicated that the CPOA hydrogel experienced cohesive failure upon peel-off (Figure S4), indicating that its mechanical properties were the primary determinant of adhesion strength. In the lap shear test, as the ratio of CP2/OAlg increased, the adhesive strength of the CPOA hydrogels first increased and then decreased, reaching a maximum when the ratio of CP2/OAlg was 5:5 (Figure 2h). Meanwhile, as the substitution degree of FPBA increased, the mechanical properties of the hydrogels improved; the adhesive strength measured for COA, CP1OA, and CP2OA was 3.65 ± 0.19, 8.24 ± 0.65, and 13.89 ± 1.52 kPa, respectively (Figure 2i), leading to a corresponding enhancement in adhesion performance.

### 3.3. ROS Responsiveness and Exos Release Behavior

#### 3.3.1. ROS Responsive Properties

**Figure 3:**
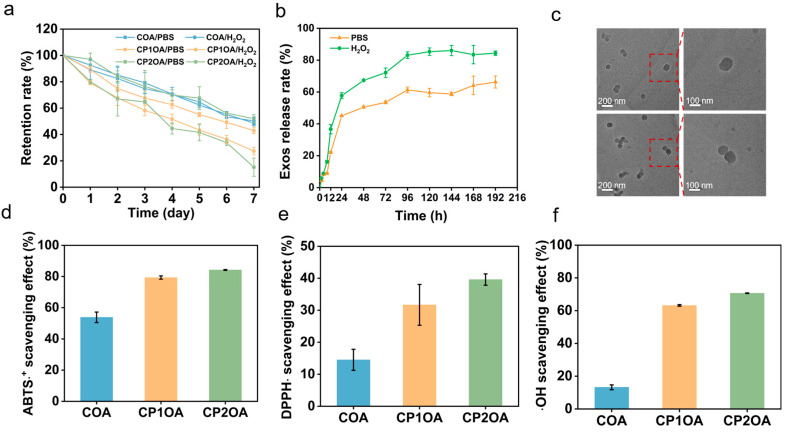
ROS responsiveness and Exos release behavior. (**a**) Degradation behaviors of CPOA hydrogels in PBS and H_2_O_2_ solution; (**b**) Exos release from CPOA hydrogels in PBS and H_2_O_2_ solution; (**c**) The Exos under TEM; Scavenging efficiency against (**d**) ABTS radical (ABTS·^+^), (**e**) DPPH radical (DPPH·) and (**f**) hydroxyl radical (·OH).

The prepared CPOA hydrogel exhibits good ROS responsiveness owing to the presence of borate ester bonds [27]. The COA hydrogel exhibited no significant difference in its retention ratio between PBS and H_2_O_2_ solution over 7 days. Specifically, its retention rates in PBS and H_2_O_2_ solution at day 7 were 50.28 ± 3.07% and 48.61 ± 2.15%, respectively. Upon introduction of a borate ester crosslinked structure, the retention rate of the CPOA hydrogel decreased in H_2_O_2_ solution, the values of CP1OA and CP2OA decreased from 43.00 ± 2.43% and 51.96 ± 3.19% in PBS to 27.45 ± 2.92% and 15.23 ± 6.92% in H_2_O_2_ solution, respectively (Figure 3a), demonstrating distinct ROS-responsive behavior. Meanwhile, the CP2OA hydrogel exhibited a faster volume reduction rate in the H_2_O_2_ solution owing to the ROS-responsive property of CP2OA (Figure S5).

#### 3.3.2. Exos Release Behavior

The higher density of borate ester bonds in CP2OA resulted in a more pronounced ROS response, thereby facilitating the responsive release of Exos. As shown in Figure 3b, the cumulative release of Exos from CP2OA hydrogel calculated through the standard curve (Figure S6a) reached 84.37 ± 1.39% in H_2_O_2_ microenvironment, significantly higher than the 66.27 ± 3.75% released in PBS over 7 days. Additionally, the Exos a spherical morphology with diameters around 100 nm (Figure 3c), and the measured nanoparticle size also falls within the range of 30–150 nm (Figure S6b), enabling their encapsulation within the hydrogel while maintaining stability.

#### 3.3.3. ROS Scavenging Ability

Given the ROS-responsive properties of the prepared CPOA hydrogels, we further evaluated their antioxidative activity. As shown in Figure 3d–f, the COA hydrogel exhibited significantly lower scavenging effect against ABTS·^+^, DPPH·, and ·OH compared with CPOA hydrogels. Furthermore, the scavenging efficiency for all three radicals was enhanced with an increase in the FPBA substitution degree, which raised the borate ester bond content within the hydrogel. CP2OA demonstrated high scavenging rates of 84.21 ± 0.27%, 39.59 ± 1.76% and 70.63 ± 0.17% against ABTS·^+^, DPPH·, and ·OH, respectively. These results indicate that CPOA hydrogels have considerable potential for mitigating excessively high ROS levels on diabetic wound surfaces.

### 3.4. In Vitro Biological Properties

**Figure 4:**
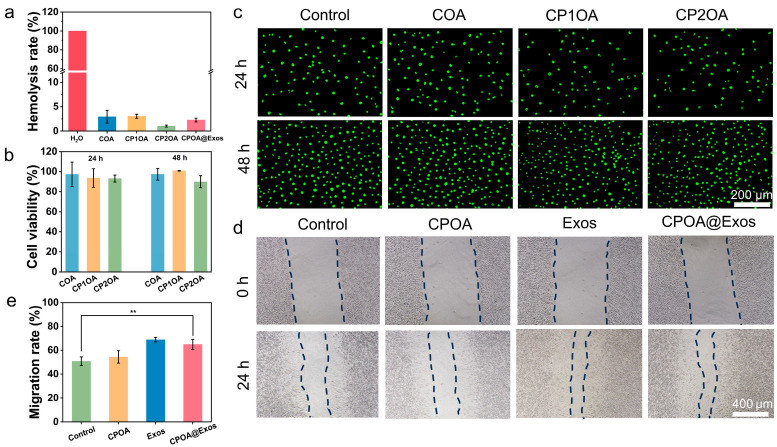
Biocompatibility and cell migratory activity of CPOA@Exos. (**a**) Hemocompatibility; (**b**) Cell viability of L929 cells assessed by CCK-8 assay; (**c**) AO fluorescence staining images of L929 cells after treatment with different hydrogels; (**d**) Cell migration images of L929 cells after co-cultured 24 h with different hydrogels or Exos; (**e**) Statistical analysis of cell migration rate in migration images. (** *p* < 0.01).

Biocompatibility was an essential requirement for the application of CPOA hydrogel in the biomedical field [18]. The CPOA@Exos system was expected to exhibit inherent biocompatibility. This was confirmed by an in vitro hemolysis assay, which showed that the hemolysis rates of all hydrogels were below 5%, indicating no significant hemolytic effect on red blood cells (Figure 4a). The cytotoxicity assay using L929 cells showed similar results. As shown in Figure 4b, after co-culturing the hydrogel extract with cells for 24 and 48 h, CCK-8 assay results suggested that there was no significant difference in cell viability compared to the control group. Similarly, AO staining also demonstrated that all cells were stained green fluorescence, and the number of cells at 48 h significantly increased compared to that at 24 h, indicating the CPOA hydrogel has no cytotoxicity (Figure 4c). Moreover, the effects of CPOA@Exos on cell migration were evaluated using a scratch assay. As shown in Figure 4d,e, the width of the scratch wound decreased noticeably over time in all groups. The cell migration rate was 50.85 ± 3.66% in the control group. A slight increase was observed in the CPOA hydrogel group (54.49 ± 5.19%), while the incorporation of Exos significantly enhanced cell migration. Specifically, the CPOA@Exos group exhibited a cell migration rate of 64.87 ± 4.23%, which was significantly higher than that of the control group (** *p* < 0.01), though slightly lower than that of the free Exos group. To investigate the effects of CPOA@Exos on the proliferation behaviors of cells, cell morphology and viability were examined at 48 h and 120 h. As shown in Figure S7a, the cell number in all groups was significantly higher at 120 h than at 48 h. At each time point, Exos and CPOA@Exos groups exhibited significantly higher cell numbers compared with the control and CPOA groups. Consistent with these observations, the CCK-8 assay confirmed that the highest proliferation rate occurred in CPOA@Exos groups (Figure S7b).

### 3.5. Repair Effects of CPOA@Exos on Diabetic Wound In Vivo

**Figure 5:**
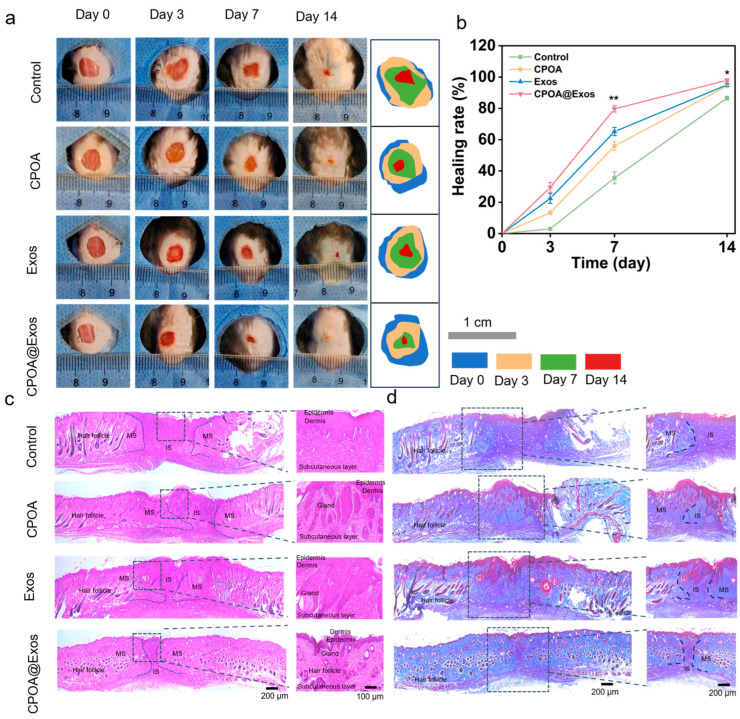
Hydrogel promoted diabetic wound healing: (**a**) Changes in wound size on days 0, 3, 7 and 14; (**b**) Wound healing rate; (**c**) H&E-stained images at day 14, immature skins (IS), mature skins (MS); (**d**) Masson’s trichrome-stained images at day 14. (* *p* < 0.05, ** *p* < 0.01).

To evaluate the repair effects of CPOA@Exos on diabetic wounds *in vivo*, an STZ-induced diabetic mouse model was established in C57 mice. Three days after STZ injection, all mice developed diabetes, characterized by sustained blood glucose levels exceeding 16.8 mmol/L, which confirmed the establishment of the model (Figure S8). After treated with different materials, the healing process was visually monitored. As shown in Figure 5a,b, the wound area decreased progressively in all groups, with all treatment groups demonstrating markedly enhanced healing rate relative to the control. On day 7, the CPOA and free Exos groups exhibited healing rates of 55.97 ± 2.82% and 65.21 ± 2.67%, respectively. Notably, the CPOA@Exos group showed a higher healing rate of 79.70 ± 2.03% than all other groups (** *p* < 0.01), approaching nearly complete healing (97.97 ± 0.90%) by day 14. This accelerated wound repair can be attributed to the complementary functions of CPOA and Exos, such as wound coverage [17], antioxidant activity [27], and anti-inflammatory activity [40]. Moreover, the ROS-responsive nature of the CPOA hydrogel enables a more controlled and prolonged release of Exos, thereby enhancing their utilization efficiency and therapeutic duration [41]. To further evaluate diabetic wound healing, histological analysis of the injured skin tissue was stained using H&E and Masson’s trichrome staining at day 14. As shown in Figure 5c, the CPOA@Exos group displayed abundant newly formed hair follicles near the wound site along with a restored and smooth epidermal surface, collectively indicating advanced tissue maturation and superior healing. Moreover, Masson’s trichrome staining (Figure 5d) revealed more collagen deposition in the CPOA@Exos group compared to other groups. During the early phase of wound healing, collagen fibers are characteristically loose and randomly oriented, whereas they gradually assemble into a dense, well-aligned architecture as tissue repair proceeds toward completion. These phenomena were consistent with the previous report [42]. Notably, the collagen fibers in the CPOA@Exos group displayed a distinctly denser and more organized arrangement relative to the control group, suggesting improved extracellular matrix remodeling and wound repair.

### 3.6. Modulation of the Diabetic Wound Microenvironment

**Figure 6:**
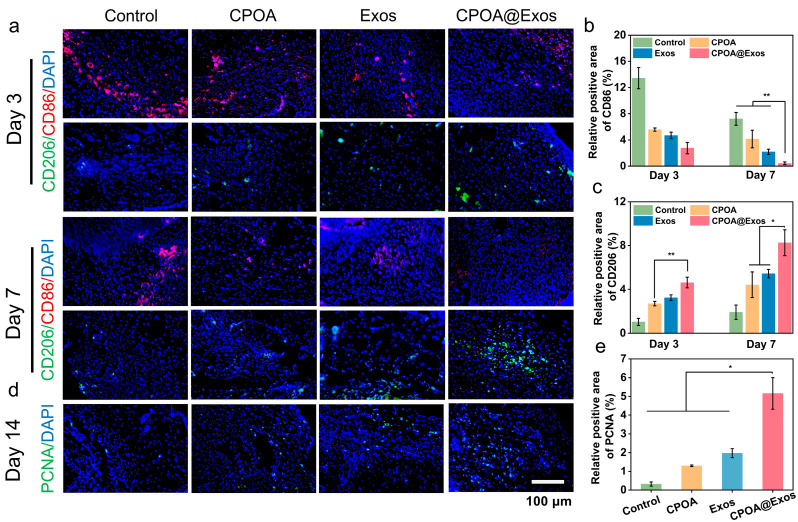
Modulation of the diabetic wound microenvironment in vivo. (**a**) Immunofluorescence staining images of CD86 and CD206 in the wound area on days 3 and 7; Statistical analysis of (**b**) CD86 and (**c**) CD206 fluorescence intensity; (**d**) PCNA immunofluorescence staining images in wound areas at day 14, (**e**) Statistical analysis of PCNA fluorescence intensity in immunofluorescence images. (* *p* < 0.05, ** *p* < 0.01).

The diabetic inflammatory response is a major factor impairing diabetic wound healing [15]. Given this, we evaluated the immunomodulatory effects of CPOA@Exos on the diabetic wound microenvironment via immunofluorescence staining for the macrophage markers CD86 and CD206. As shown in Figure 6a, CD86-labeled pro-inflammatory M1 phenotype macrophages (red) were predominant in the control group on day 3, with only a sparse presence of CD206-labeled anti-inflammatory M2 phenotype macrophages (green). The control group exhibited severe inflammatory responses, with macrophages predominantly polarized into the M1 phenotype characterized by phagocytic and clearance properties, showing a CD86 expression rate of 13.44 ± 1.61%. Notably, M1 macrophages remained the dominant population (7.22 ± 0.97%) even by day 7 (Figure 6b). Furthermore, due to the good antioxidant capacity of the CPOA hydrogel and the immunomodulatory function of Exos, the CD86-positive cell population in both CPOA and Exos groups was significantly decreased compared to the control group on both days 3 and 7. A corresponding increase was observed in the expression levels of CD206-positive cells. As expected, the CPOA@Exos group demonstrated the lowest CD86 expression level of 2.77 ± 0.86% on day 3 and 0.45 ± 0.22% on day 7 (* *p* < 0.05, ** *p* < 0.01), alongside the highest CD206 expression levels (4.63 ± 0.50% and 8.25 ± 1.18% on days 3 and 7) (Figure 6c). At the same time, CPOA@Exos effectively upregulated the anti-inflammatory cytokine IL-10 (Figure S9a,b) and downregulated the pro-inflammatory cytokine TNF-α (Figure S9c) compared to the control group, thereby suppressing excessive inflammatory responses in the wound microenvironment.

These results suggest a phenotypic shift in macrophages within the wound tissue, towards the anti-inflammatory M2-phenotype macrophages, which are known to secrete factors that promote angiogenesis and resolve inflammation. This transition indicates that CPOA@Exos can effectively modulate macrophage polarization to M2 during the inflammatory phase of wound healing. Owing to its modulatory effect on the inflammatory microenvironment, the developed CPOA@Exos hydrogel can further regulate cellular proliferation in the injured area. As shown in Figure 6d,e, the number of PCNA-positive cells in CPOA and Exos increased compared with the control group, and the number of PCNA-positive cells in CPOA@Exos was the highest compared with other groups.

## 4. Conclusions

In summary, this study fabricated a hydrogel by grafting FPBA onto CMCS and mixed it with OAlg, resulting in a dynamic crosslinked network based on Schiff base and borate ester bonds. The hydrogel can effectively load exosomes. The prepared CPOA hydrogel demonstrated stable mechanical strength, tissue adhesion, and ROS responsiveness. Moreover, the dual-crosslinked hydrogel enhanced multiple hydrogel characteristics, including suitable gelation time, self-healing ability, injectability, mechanical robustness, and adhesiveness. Leveraging dynamic borate ester bonds, it enabled sustained release of exosomes at the diabetic wound site while effectively scavenging excess ROS, thereby facilitating the healing process. The CPOA@Exos system was shown to modulate inflammatory responses in diabetic wounds, promote macrophage polarization toward the M2 phenotype, and accelerate wound repair. This work presents a promising therapeutic strategy for the management of diabetic wounds.

## Data Availability

The original contributions presented in this study are included in the article/Supplementary Material. Further inquiries can be directed to the corresponding author.

## Supplementary Materials

The following supporting information can be downloaded at: https://www.mdpi.com/article/10.3390/ma19020445/s1, Table S1: The formulations of hydrogels. Figure S1. Morphology of COA, CP1OA and CP2OA hydrogel, Figure S2: Rheological properties of the hydrogels: (a) Strain amplitude sweeps test of hydrogels with different ratios; (b) Strain amplitude sweeps test of COA, CP1OA and CP2OA; (c) Shear-thinning behavior of hydrogels with different ratios, Figure S3: Pictures of self-healing and adhesion of hydrogels: (a) Self-healing process of hydrogels; (b) Adhesive performance of the hydrogels on various substrates, Figure S4: The adhesion stress of COA, CP1OA and CP2OA hydrogel via bonding test, Figure S5: Morphological and pH value changes of CP2OA immersed in PBS and H_2_O_2_ within 7 days, Figure S6: Exos characterization: (a) The standard curve of Exos assessed by BCA kit; (b) Particle size of the Exos, Figure S7. Cell proliferative behaviors in the presence of CPOA@Exos. (a) Cell morphology at 48 h and 120 h; (b) Cell viability rate at 48 h and 120 h (* *p* < 0.05, ** *p* < 0.01), Figure S8: Changes in blood glucose in mice after injection of STZ, Figure S9: Immunohistochemical staining images of diabetic wounds on day 7 after treatment by CPOA@Exos. (a) Immunohistochemical images of IL-10 and TNF-α; (b) Positive area ratio of IL-10; (c) Positive area ratio of TNF-α (* *p* < 0.05, ** *p* < 0.01).
