# Supplementary material for: A Dual-Dynamic Crosslinked Polysaccharide-Based Hydrogel Loaded with Exosomes for Promoting Diabetic Wound Healing
Source: Materials (Basel). 2026 Jan 22;19(2):445. doi: 10.3390/ma19020445 (PMC12842903; doi:10.3390/ma19020445)
Supplement: Supplementary file 1 [file materials-19-00445-s001.zip › materials-4045305-supplementary.pdf]

# Supporting Information

# A Dual-Dynamic Crosslinked Polysaccharide-Based Hydrogel Loaded with Exosomes for Promoting Diabetic Wound Healing

**Ding Lin[1], Zhenhao Li[1], Jianying Hao[1], Xiaobo Xu[1], Xiuqiang Li[1,2], Yuan Feng[1], Xiaochen Lu[1], Fanglian Yao[1,3,4], Hong Zhang[1,3,4] and Junjie Li[1,3,4,*]**

[1] School of Chemical Engineering and Technology, Tianjin University, Tianjin 300350, China; abc120010_@tju.edu.cn (D.L.)

[2] School of Science, Tianjin University, Tianjin 300350, China

[3] State Key Laboratory of Synthetic Biology, Tianjin University, Tianjin, 300350, China

[4] Frontiers Science Center for Synthetic Biology (Ministry of Education), Tianjin University, Tianjin, 300350, China

* Correspondence: li41308@tju.edu.cn;

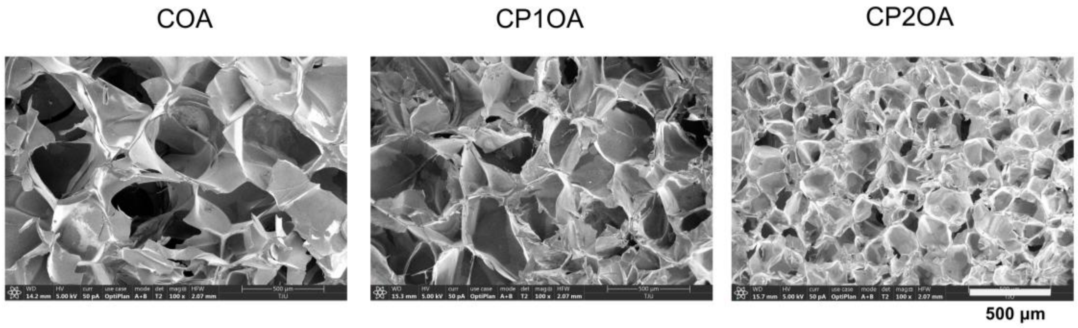


**Figure S1.** Morphology of COA, CP1OA and CP2OA hydrogel.

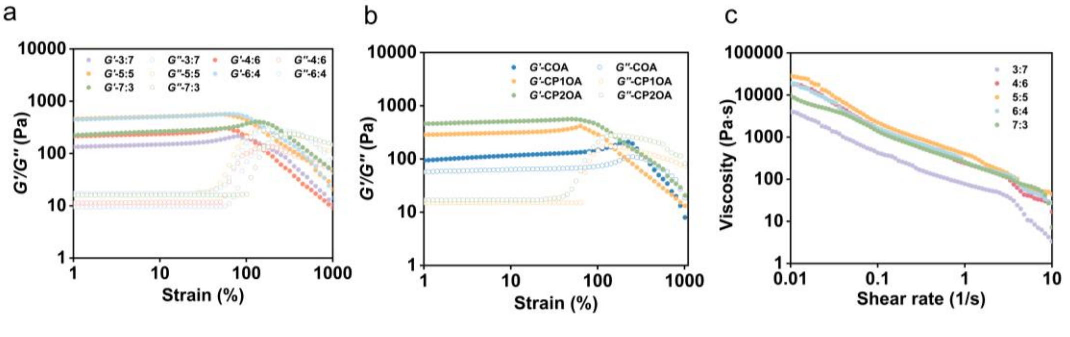


**Figure S2.** Rheological properties of the hydrogels: (**a**) Strain amplitude sweep tests of hydrogels with different ratios; (**b**) Strain amplitude sweep tests of COA, CP1OA and CP2OA; (**c**) Shear-thinning behavior of hydrogels with different ratios.

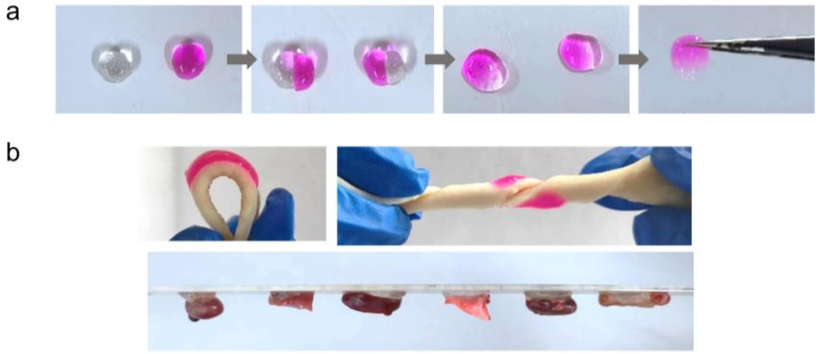


**Figure S3.** Pictures of self-healing and adhesion of hydrogels: (**a**) Self-healing process of COPA hydrogels; (**b**) Adhesive performance of the hydrogels on various substrates.

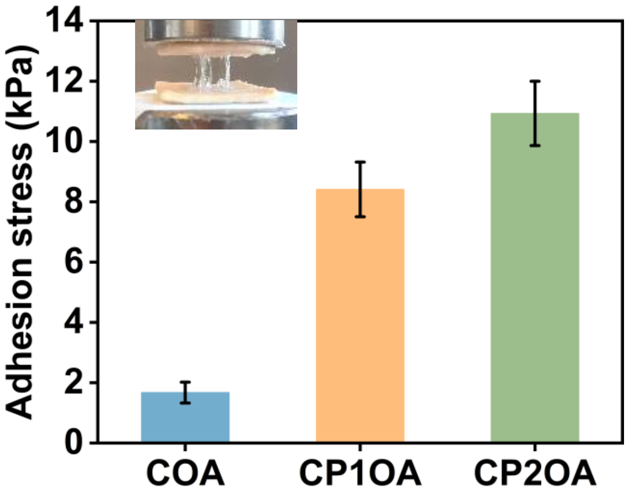


**Figure S4.** The adhesion stress of COA, CP1OA and CP2OA hydrogel via bonding test.

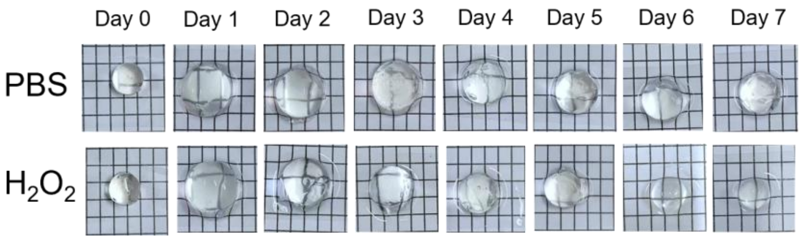


**Figure S5.** Morphological changes of CP2OA immersed in PBS and $H_2O_2$ within 7 days.

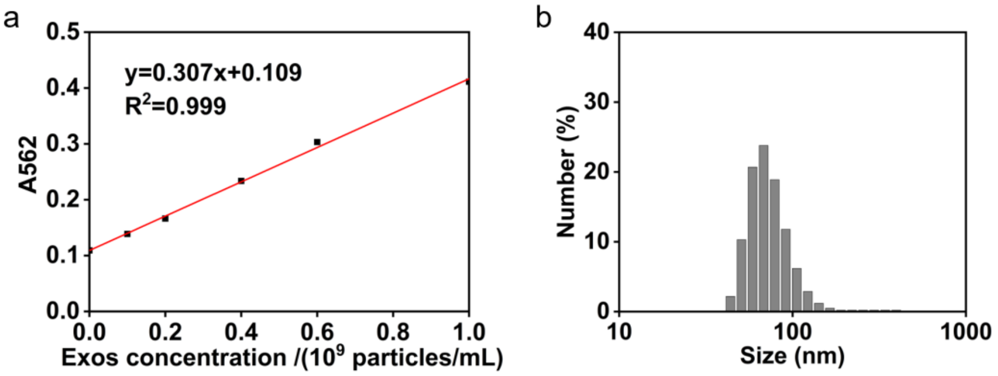


**Figure S6.** Exos characterization: (**a**) The standard curve of Exos assessed by BCA kit; (**b**) Particle size of the Exos.

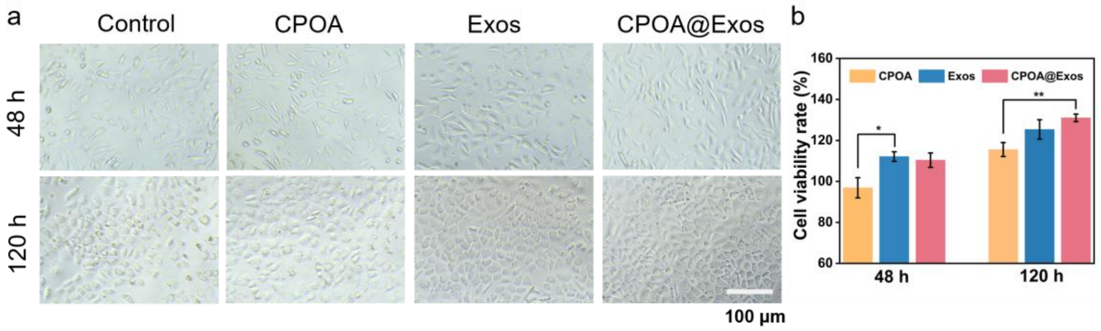


**Figure S7**. Cell proliferative behaviors in the presence of CPOA@Exos. (**a**) Cell morphology at 48 h and 120 h; (**b**) Cell viability rate at 48 h and 120 h. (* $p < 0.05$, ** $p < 0.01$).

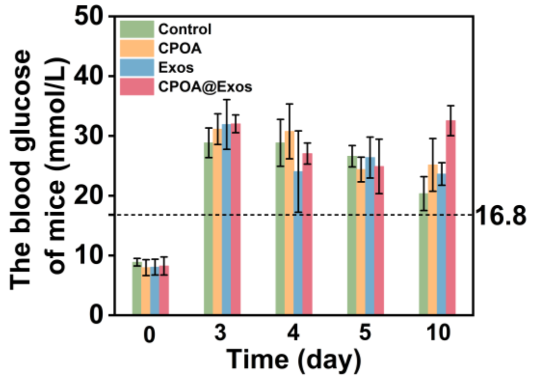


**Figure S8.** Changes in blood glucose in mice after injection of STZ.

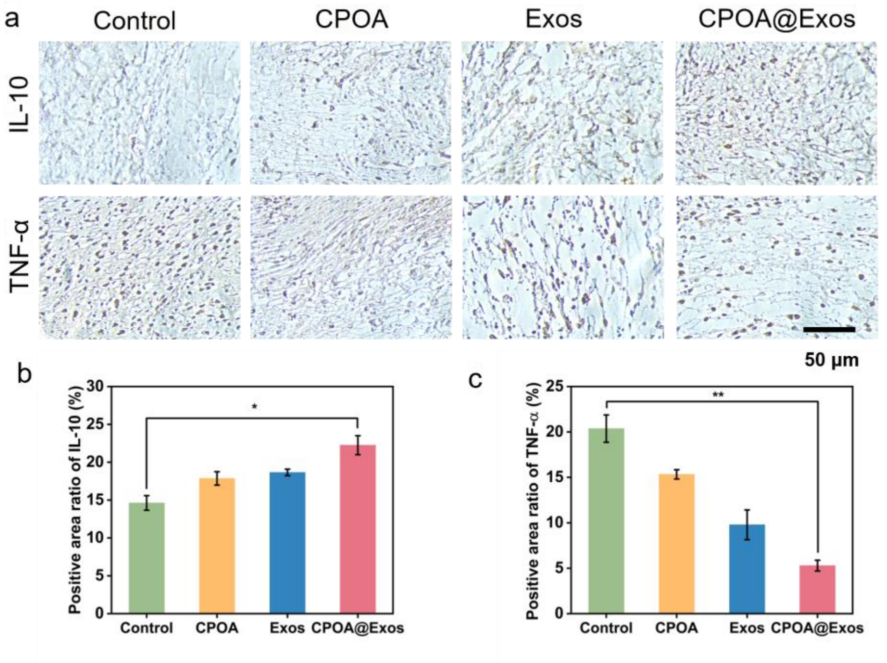


**Figure S9.** Immunohistochemical staining images of diabetic wounds on day 7 after treated by CPOA@Exos. (**a**) Immunohistochemical images of IL-10 and TNF-$\alpha$; (**b**) Positive area ratio of IL-10; (**c**) Positive area ratio of TNF-$\alpha$. (* $p < 0.05$, ** $p < 0.01$).

**Table S1.** The formulations of hydrogels.

| | $H_2O$/mL | OAlg/g | CMCS/g | CP1/g | CP2/g | Exos ($10^{10}$ particles/mL) |
|---|---|---|---|---|---|---|
| COA | 10 | 0.6 | 0.6 | | | |
| CP1OA | 10 | 0.6 | | 0.6 | | |
| CP2OA | 10 | 0.6 | | | 0.6 | |
| CP2OA@Exos | 9 | 0.6 | | | 0.6 | 1 |
